# Annotations

**Published:** 1892-06-04

**Authors:** 


### Annotations.

**Home Life for Working Girls.**

Every woman wants a home; it is an instinct with her even more than with a man; and one reason why girls shrink so much from going out into the world, even when it would obviously be to their advantage in other ways, is this dread of being homeless. For many working girls lodgings are as cheerless as they are dear, and from sheer loneliness they drift into company that is frivolous even when it is not bad. It is to remedy this that the Homes for Working Girls have been established in various districts of London. There are now nine of them, occupied by girls of all classes, from domestic servants to governesses. A large number are shop girls or workers in large dressmaking and millinery establishments. For four shillings a week they can have a neat cubicle, and for half-a-crown a bed in a dormitory with six or eight others. Food costs four and sixpence a week, and in all the nine establishments there has never been a complaint about it. Girls are not received if they are more than twenty-five years of age, for the accommodation is not unlimited, and women of that age may reasonably be supposed to be able to protect themselves from the dangers into which their younger sisters might in their ignorance fall. Still, looking at the clean, pleasant chambers, and thinking of the moderate rent, one cannot but regret that they are not open to all. The homes are not fully self-supporting, or this might be granted; but though the girls' payments cover rent, food, and servants' wages, subscriptions must be sought to pay for repairs and other incidental expenses. It is pleasant to see, however, that those who obtain admission value it, and this may be inferred from the little treasures — books, flowers, illuminated texts — with which they almost all adorn their little nests. In the great hive of London it is pleasant to see such provision for the most defenceless of the working bees, and since their wages are not large enough in many cases to keep them entirely, those who like to help women to be independent might well subscribe to these homes. The treasurer is Lord Kinnaird, 10, St. James's Square, W., and all information can be had from the honorary director, Mr. John Shrimpton, Westminster Chambers, 3, Victoria Street, S.W.

**A Premium on Inefficiency.**

We have often urged in these columns the importance of maintaining the voluntary system of hospital support. We have proved, over and over again, that our voluntary hospitals are, on the whole, probably the most efficiently administered institutions in the country. This efficiency is largely due to the fact that every voluntary hospital is necessarily open to the public. Most things which happen within the walls, certainly everything of a discreditable character, is sure, ultimately, to be made public. In rate-supported institutions in this country publicity is avoided. Any ratepayer who went as a private citizen and demanded entrance on the ground that he wished to see how the institution was managed would most certainly be refused admission. In many rate-supported institutions there is little or no clinical instruction, and the whole administration is practically hidden from the public gaze. Strong evidence and a proof of the danger of this condition of affairs, and of its injustice, is supplied by a circumstance connected with the recent enquiry into the Eastern Hospital scandal, for which we are indebted to Mr. Simpkins, a working man. He was so appalled by the mismanagement of this rate-supported institution during ten weeks' residence within it as a patient that he set himself to work to procure an official enquiry. In this public-spirited act he was successful, and thanks to him the Eastern Hospital is likely for the future to be managed with some regard to efficiency and economy. Will it be credited that Mr. Simpkin has now been called upon to pay £136, being the costs of promoting this successful enquiry on behalf of the public, whilst the hospital authorities spent upwards of £900 on their defence, which latter sum the ratepayers have had to pay? An appeal to the Local Government Board and the Lord Chancellor proved ineffectual, and the *Hackney Mercury* has therefore instituted a subscription to refund the £136 which Mr. Simpkin, despite his large family, has been called upon to pay. We hope that many of our readers will be moved to send a subscription towards this expense, which we shall be glad to take charge of and to forward in due course to the proper authorities. Probably nothing more monstrous than the fate of Mr. Simpkin has happened in the history of rate-supported hospitals since the world began. It is perfectly certain that the whole of the costs ought to be charged to the incriminated parties whose gross mismanagement the enquiry brought to light.

**Mr. Egmont Hake's Book.**

Mr. Egmont Hake's book on "Suffering London," has been noticed at varying length by the leading newspapers; and the notice, we may hope will bear fruit abundantly. It must not be considered that there is anything final either in the book itself or in its issue as the beginning of a forward movement on the part of hospitals. It is but the first step, the unfurling of the flag; and it must be intelligently and resolutely followed up by other effective action. It is a great thing that the hospitals have come to see and acknowledge the value of concerted action. If they continue to act together, they cannot fail to make an abiding impression upon the intelligence of the country. Two main ideas must be kept in mind: First, that the hospitals are a most important and indispensable branch of the public service; and second, that if they are to continue to be maintained by voluntary gifts, it can only be by the voluntary gifts of the community as a whole—not by the gifts of a few. The impressing of these ideas upon all classes must be the work of the immediate future; and it is a work which can no more be done in a day than Rome was built in a day. Voluntaryism in hospitals is now put upon its mettle; but there can be no doubt at all about the result if the campaign be conducted with an adequate view of its importance, and with wisdom and persevering energy. There is a vast amount of new territory to be conquered before the voluntary gifts of charity will be adequate and continuous. But it is in the character of the English people themselves that we find the best hope of success. They are for the most part quite willing to be conquered, quite willing to be converted to the hospital faith. But if conversions are to be as numerous and as genuine as we hope, it will be necessary to act wisely in the choice of missionaries and apostles for the advocacy of our cause. Where a prudent and resourceful Wellington would succeed, an impulsive Monmouth will almost certainly fail. It is imperative for all of us at the present time to recognise the magnitude of the work still to be done, and that in the publication of Mr. Egmont Hake's book, the first step alone has been taken. If unanimity and intelligence continue to mark the conduct of hospital workers, there are no difficulties in the path which may not be effectually overcome.

